# Physical Activity and Inhibition of ACE Additively Modulate ACE/ACE-2 Balance in Heart Failure in Mice

**DOI:** 10.3389/fphar.2021.682432

**Published:** 2021-06-07

**Authors:** Urszula Tyrankiewicz, Mariola Olkowicz, Piotr Berkowicz, Magdalena Jablonska, Ryszard T. Smolenski, Jerzy A. Zoladz, Stefan Chlopicki

**Affiliations:** ^1^ Jagiellonian Centre for Experimental Therapeutics (JCET), Jagiellonian University, Krakow, Poland; ^2^ Department of Biochemistry, Medical University of Gdansk, Gdansk, Poland; ^3^ Department of Magnetic Resonance Imaging, Institute of Nuclear Physics, Polish Academy of Sciences, Krakow, Poland; ^4^ Department of Muscle Physiology, Faculty of Rehabilitation, University School of Physical Education, Krakow, Poland; ^5^ Chair of Pharmacology, Jagiellonian University Medical College, Krakow, Poland

**Keywords:** heart failure, angiotensins, angiotensin-converting enzyme inhibition, spontaneous physical activity, mice

## Abstract

Angiotensin-converting enzyme inhibition (ACE-I) and physical activity favorably modulate the ACE/ACE-2 balance. However, it is not clear whether physical activity and ACE-I could synergistically modulate ACE/ACE-2 balance in the course of heart failure (HF). Here, we studied the effects of combined spontaneous physical activity and ACE-I–based treatment on angiotensin (Ang) pattern and cardiac function in a mouse model of HF (Tgαq*44). Tgαq*44 mice with advanced HF (at the age of 12 months) were running spontaneously in a running wheel (exercise training group, ExT) and/or were treated with ACE inhibitor (ACE-I, perindopril, 10 mg/kg) for 2 months. Angiotensin profile was characterized by an LC-MS/MS-based method. The cardiac performance was assessed *in vivo* by MRI. Ang-(1–7)/Ang II ratio in both plasma and the aorta was significantly higher in the combined treatment group than the ACE-I group or ExT alone, suggesting the additive favorable effects on ACE-2/Ang-(1–7) and ACE/Ang II axes’ balance induced by a combination of ACE-I with ExT. The basal cardiac performance did not differ among the experimental groups of Tgαq*44 mice. We demonstrated additive changes in ACE/ACE-2 balance in both plasma and the aorta by spontaneous physical activity and ACE-I treatment in Tgαq*44 mice. However, these changes did not result in an improvement of failing heart function most likely because the disease was at the end-stage. Ang-(1–7)/Ang II balance represents a valuable biochemical end point for monitoring therapeutic intervention outcome in heart failure.

## Introduction

Pharmacotherapy of heart failure (HF) delays the progression of the disease but if combined with lifestyle modifications, including exercise training (ExT) could be more effective (Piepoli, 2013; Arnett et al., 2019). Inhibitors of renin–angiotensin–aldosterone system (RAAS) constitute a cornerstone of therapy of HF and the efficacy of angiotensin-converting enzyme inhibitors (ACE-Is), angiotensin II receptor blockers (ARBs), and finally, mineralocorticoid receptor antagonists (MRAs) have been firmly established. RAAS blockade inhibits neurohormonal overactivity in HF and favorably changes the balance of ACE/Ang II and ACE-2/Ang-(1–7) pathways toward the latter one. Similarly, spontaneous physical activity activates mechanisms that promote the vasoprotective and cardioprotective ACE-2/Ang-(1–7) pathway (Wang et al., 2019), although possibly this acts through different mechanisms (Zheng et al., 2012; Zucker et al., 2015), thus may have additional effects on top of pharmacological RAAS blockade. In fact, the combined effect of pharmacotherapy (ACE-I) and exercise training was observed in several studies as evidenced by a decreased mortality and a decreased rate of hospitalization in HF patients, including patients with ischemic heart disease (O’Connor et al., 2009; Kabboul et al., 2018). However, based on the results of one of the largest multicenter, randomized controlled trials of exercise training in HF (HF-ACTION), the significance of combined therapy on patients’ mortality and rate of hospitalization was nonsignificant or (after adjustment for strongly predictive factors) had only a modest effect (O’Connor et al., 2009). Other clinical studies supported beneficial effects of combined therapy (pharmacological and exercise) including not only ACE-Is but also AT_1_R blockers, beta-antagonists, or others (Anderson et al., 2016; Abell et al., 2017). These clinical results, however, referred to various programs of physical activities (with various training frequency of 1–7 per week, length of each training sessions varied between 20 and 90 min/session, and with different training intensity) that could explain why some studies confirmed improvement in cardiac performance by combined treatment with ACE-I and exercise (Baptista et al., 2018) while others did not (Sullivan et al., 1988; Belardinelli et al., 1999; van Tol et al., 2006). In experimental studies, which explored the effect of ACE-I combined with ExT, some of them showed potentiated effect on the cardiac structure or cardiac performance (Ziada et al., 2005; Ziada, 2009), and other authors did not confirm additive impact of the combined therapy (Felix et al., 2018) on cardiac performance.

Accordingly, while effects of ACE inhibition in HF is well described, the effects of physical activity are often dependent on implementation of various physical training programs, with various end point parameters of combined therapies referring to mortality and rate of hospitalization (O’Connor et al., 2009; Abell et al., 2017) but not to mechanistically relevant biochemical biomarkers. While the effect of combined therapy on patients’ mortality and rate of hospitalization or quality of life are of key importance, the precise mechanisms and factors playing role in above end points remain not fully elucidated. It is evident that both ACE-I and regular exercise regulate the key RAAS elements [such as Ang II (Swedberg et al., 1988; Nunes-Silva et al., 2017), but there is no unequivocal evidence about how the combined therapy affect the counter-regulatory RAAS axis [ACE-2/Ang-(1–7)] and other important RAAS components and their metabolites [e.g., Ang-(1–9), Ang III, IV, and alamandine].

In fact, the lack of reliable biochemical end point in these interventional studies represents an important limitation to confirm and to guide the therapeutic application of the combination of exercise and ACE blockade.

Our goal was fill this gap and to characterize the effect of therapy involving ACE-I or/and ExT on a broad spectrum of angiotensin (Ang) peptides in relation to cardiac function in the course of HF to find out whether Ang-(1–7)/Ang II ratio could serve as a reliable and mechanistically-relevant biomarker. For that purpose, we measured angiotensin II or Ang-(1–7) and several others angiotensins at the systemic (plasma) and local (aorta) level and monitor changes in their concentration induced by single or combined therapies (ACE-I and ExT).

For that purpose, we used a unique model of murine HF that develops HF progressively starting from the age of 2–4 months till the end-stage HF at the age of 12–14 months (Mende et al., 2001; Tyrankiewicz et al., 2018). A special feature of the Tgαq*44 murine model is that the overexpression of an active αq subunit of the G protein selectively in cardiomyocytes mimics persistent receptor (adrenergic α_1_, angiotensin AT_1_, and endothelin ET_A_) stimulation and activates the intracellular signal pathways which are known to play a key role in human HF. Consequently, the Tgαq*44 mice model mimics human HF at the molecular, biochemical, and functional level. At about 2–4 months of age in Tgαq*44 mice, cardiac performance is still relatively preserved, but cardiomyocyte hypertrophy, and remodeling of the extracellular matrix (Édes et al., 2008; Mackiewicz et al., 2012) together with the activation of hypertrophic myocardial genes (ANP, BNP, and MHC-β) are all present (Mende et al., 2001; Drelicharz et al., 2009). Progressive cardiac functional deterioration in Tgαq*44 mice (Tyrankiewicz et al., 2018) associated with the downregulation of PKA signaling and changes in myofilament protein phosphorylation, culminating with the end-stage HF phenotype goes hand in hand with the activation of the classic RAAS pathway, which leads to significant imbalance in the ACE/ACE-2 axis—a typical hormonal abnormality observed in human HF patients. Along with the cardiac functional deterioration in Tgαq*44 mice, the mitochondrial function as well as systemic physical performance is impaired (Elas et al., 2008; Grassi et al., 2017; Bardi et al., 2019), and finally, the heart failure phenotype observed at the 12–14 months of age mimics the human end-stage of cardiac pathology and is characterized by high mortality and is associated with profoundly enlarged chambers, significant fibrosis, pulmonary edema, loss of cardiac inotropic, lusitropic and chronotropic reserve, and impairment of NO-dependent coronary vasodilation and increased oxidant stress (Drelicharz et al., 2009; Mackiewicz et al., 2012; Proniewski et al., 2018; Tyrankiewicz et al., 2018). Interestingly*,* platelet hyperreactivity and brain endothelium inflammatory activation were also detected in this model at the early HF stages, together with the vascular cognitive impairment (Adamski et al., 2018). Altogether, slow development of cardiac (initially) and systemic consequences of cardiac insult mimic chronic HF phenotype observed in humans, which makes the Tgαq*44 model particularly suitable for studies regarding pathophysiology and therapeutic intervention in HF. Several therapies were applied to Tgαq*44 mice including ACE-I–based pharmacotherapy and spontaneous running with positive effects of both of them on cardiac performance and HF progression (Mende et al., 2001; Tyrankiewicz et al., 2013; Wozniak et al., 2013; Grassi et al., 2017; Bardi et al., 2019).

Our previous studies demonstrated that at the advanced stage of HF in Tgαq*44 mice, when ejection fraction (EF) was significantly decreased and cardiac reserve was lost (in 12-month -old mice) (Tyrankiewicz et al., 2018), only modest improvement in cardiac performance was possible following 2 months of ACE-I treatment combined with aldosterone receptor antagonist mostly related to attenuation of LV remodeling and preservation of functional reserve, without any significant effects on myocardial function parameters at rest (Wozniak et al., 2013). In contrast, at the early stage of HF, RAAS inhibition improves resting myocardial function in Tgαq*44 mice. Similarly, ExT alone inhibited HF progression in younger but not older Tgαq*44 mice with advanced HF (Bardi et al., 2019). These data suggest that Tgαq*44 mice represent a relevant model of HF progression with RAAS overactivity responsible for HF progression (Wozniak et al., 2013; Tyrankiewicz et al., 2018). Furthermore, ACE/ACE-2 ratio seemed to be an important determinant of the HF progression in Tgαq*44 mice (Tyrankiewicz et al., 2018). Here, we tested the hypothesis that combined therapy (ACE-I treatment and ExT) in the advanced stage of HF would have an additive effect on ACE/ACE-2 balance in HF progression in Tgαq*44 mice and this response would be translated into better cardiac performance.

## Materials and Methods

### Animals

Transgenic, homozygous female Tgαq*44 mice (bred on FVB mice background), characterized by cardiac-specific overexpression of activated Gαq protein, that mimic well human HF phenotype by chronic development of the left ventricle (LV) hypertrophy and dilatation, cardiac fibrosis, and inflammation (Mende et al., 2001) were used here as well as age-matched wild-type control mice (FVB). All animal procedures (including handling) conformed to the Guide for the Care and Use of Laboratory Animals published by the U.S. National Institutes of Health (NIH Publication No. 85-23, revised 1996), Guidelines for Animal Care and Treatment of the European Community, and to the local Ethical Committee on Animal Experiments in Krakow (agreement no: 27/2014). 12-month-old Tgαq*44 mice used here represent phenotype of advanced HF and are characterized by a decreased EF, lack of cardiac reserve and profoundly reduced cardiac function, and impaired physical activity (Elas et al., 2008; Czarnowska et al., 2016; Grassi et al., 2017; Tyrankiewicz et al., 2018; Bardi et al., 2019). Tgαq*44 mice were randomly divided into exercised and sedentary group (ExT and Sed, respectively), and each sedentary and exercised group was then divided into control (*N* = 7 for sedentary and *N* = 12 for exercised group) or ACE-I–treated group (perindopril, Sigma-Aldrich, 10 mg/kg daily in drinking water; *N* = 6 for sedentary and *N* = 9 for the exercised group). The dose of perindopril was chosen based on previous studies in this model where we demonstrated that 10 mg/kg in drinking water was effective to inhibit HF progression in Tgαq*44 mice at the relatively early stage of the disease (Wozniak et al., 2013). The healthy wild-type control, age-matched FVB mice were divided into sedentary and exercised groups (*N* = 6 and *N* = 11, respectively). Each mouse from the ExT group was located separately in the cage with a running wheel for a period of 8 weeks. Tgαq*44 mice were less active on the running wheel (189.6 ± 49.3 h) and covered shorter distances (189.4 ± 70.4 km) than FVB mice (358.5 ± 70.7 h; 540.5 ± 133.6 km, respectively) that was not influenced by ACE-I treatment resulting in the following run times/distances: 205.8 ± 63.9 h; 198.9 ± 100.9 km, respectively, for the ExT/ACE-I–treated Tgαq*44 group. These results, in accordance with our previous publications (Grassi et al., 2017; Tyrankiewicz et al., 2018), confirmed reduced running performance of Tgαq*44 mice as compared to wild-type FVB mice and much declined in old *vs.* young Tgαq*44 mice.

### Evaluation of Balance Between ACE–Ang II–AT_1_R and ACE-2–Ang (1–7)–Mas Axes Existing in Plasma and Aortic Tissue Compartments by Comprehensive Angiotensin Profiling.

Mice were injected with heparin, then with ketamine and xylazine (100 and 10 mg/kg, respectively). After the onset of anesthesia, the blood was drawn from the renal artery and collected in tubes containing EDTA (25 μg/ml). The samples were taken only from mice which passed all procedures experimental time (8 weeks + MRI scans). Some of mice died during 8 weeks of experimental time or under MRI scanning probably because of advanced HF (reduced EF and ECG perturbations) that was also observed in the sedentary mice group with advanced HF. Samples were immediately mixed with protease inhibitor cocktail [(Sigma-Aldrich) in a ratio of 19:1 (v/v)] and centrifuged at 2,000×*g* for 15 min to isolate plasma. After the centrifugation, the resulting plasma was transferred into the Protein LoBind tubes (Eppendorf, Hamburg, Germany), split into aliquots, and stored at −80°C until further sample preparation. Thoracic aortas were isolated, cleaned from surrounding tissues in cold 0.9% saline solution, and preserved for further analysis.

Protein precipitation with acetonitrile (ACN) followed by C18-based solid-phase extraction (SPE) was applied for Ang peptide extraction from both specimens analyzed and to remove high-molecular plasma/aortic tissue compounds. Briefly, plasma samples were deproteinized with ACN that was employed in 4:1 (vol/vol) proportion to the samples used and further subjected to cleanup procedure with C18 silica-based SPE cartridges. In turn, to obtain the protein tissue extracts, aortas were homogenized on ice in a 0.9% saline/0.1 mol/l HCl solution supplemented with 1% (vol/vol) protease inhibitor cocktail (Sigma-Aldrich). The total protein content in aliquots of homogenates collected was determined every time with BCA protein assay kit (Novagen; Merck Millipore). Relevant peptides’ extraction (from tissue specimens) was performed as outlined above. More details regarding sample pretreatment procedure applied can be found in (Olkowicz et al., 2017).

All LC-MS/MS-based angiotensin measurements were performed as previously described (Olkowicz et al., 2017; Tyrankiewicz et al., 2018). Briefly, an UltiMate 3000 Rapid Separation nanoLC system (Dionex; Thermo Scientific) interfaced *via* a ChipMate nanoelectrospray ion source (Advion) to a TSQ Vantage triple quadrupole mass spectrometer (Thermo Scientific) was used. The samples were injected in 5 µl aliquots onto a trapping column for desalting and concentrating of the analytes that were further switched in line with the separation column to elute the peptides at a flow rate of 300 nl/min with an increasing percentage of the organic solvent. Chromatographic separation was accomplished using a gradient of phase A [acetic acid (1%, vol/vol) in H_2_O] and phase B [acetic acid (1%, vol/vol) in ACN] as follows: 2% of phase B for 5 min, 2–98% of phase B for 15 min, and 98% of phase B for 5 min, resulting in a total run time of 40 min together with the re-equilibration. Data acquisition was carried out in the selected reaction monitoring (SRM) mode employing electrospray positive ionization mode. Two ion transitions were monitored for endogenous angiotensin peptides [Ang I, II, III, IV, (1–7), (1–9), Ang A, alamandine, and Ang-(1–12)] as well as internal standard—[Asn^1^ and Val^5^]-Ang II. Detailed analytical conditions, as well as the precursor and product ions used in the protocol, have been described previously (Olkowicz et al., 2017). The LC/MS system, data acquisition, and processing were managed by Xcalibur software (version 2.1; Thermo Scientific).

### Measurements of Cardiac Function by MRI *in vivo*

After 8 weeks of treatment, MRI cardiac *in vivo* experiments were performed using a 9.4T scanner (BioSpec 94/20 USR, Bruker, Germany) with high-performance gradient coils and 36 mm 1H quadrature volume resonator, according to the protocol described previously (Tyrankiewicz et al., 2018). Bright blood cine images of the heart were acquired with retrospectively gated IntraGateFLASH sequence (echo time TE 1.5 ms, repetition time TR 4.3 ms, flip angle (FA) 17°, field of view (FOV) 30 × 30 mm^2^, matrix size 192 × 192 reconstructed to 256 × 256, slice thickness 1.0 mm, and 300 repetitions). ParaVision5.1 macro IntraGate was used for image reconstruction (up to 60 frames per cardiac cycle). Three long axes: 2-, 3-, and 4-chamber (2CH, 3CH, and 4CH) and contiguous short-axis (SAX) images covering the entire LV cavity were acquired. Dobutamine test SAX images were positioned at the mid-cavity LV level. Mice were anesthetized with isoflurane (1.7% in oxygen and air mixture), and their ECG, respiration, and body temperature (maintained at 37°C by water heating) were monitored (SA Instruments Inc., Stony Brook, United States). The concentration of anesthetic drug was strictly controlled and maintained at the same level throughout all measurements as isoflurane might influence cardiac perfusion (Kober et al., 2005; Berry et al., 2009). Cardiac function was assessed in regard to LV end-systolic and end-diastolic chamber volume, stroke volume, cardiac output, heart rate, ejection rate, filling rate, and isovolumic relaxation and contraction time. Dobutamine (Sigma-Aldrich, United States) at a low and high dose (0.5 and 2 mg/kg, i.p*.*, respectively) was injected for the assessment of cardiac reserve (measured from 1 mm slice at the papillary muscle level of LV). Then, the maximal cardiac response was taken for analysis. Time intervals were assessed from the cardiac cycle for the assessment of the isovolumic phase, ejection and filling durations, and myocardial performance index, as described previously (Tyrankiewicz et al., 2018).

### Statistical Analysis

Statistical analyses were performed using GraphPad Prism 8 (GraphPad Software) software. Results are presented as mean ± SD (or in case of peptides’ assessment as mean ± SEM). Exercise performance between Tgαq*44 and FVB mice was verified with one-way ANOVA test. Basal cardiac function for FVB mice (sedentary *vs.* exercised) as well as for control groups (healthy *vs.* HF: FVB *vs.* Tgαq*44 mice) and the response to dobutamine for each group (basal cardiac function vs. cardiac function under stress conditions) was assessed with *t* test. Normality of distribution and homogeneity of variance were tested using the Shapiro–Wilk test and Brown–Forsythe test, respectively. When these assumptions were not satisfied, the nonparametric tests were performed (the Wilcoxon test for paired groups and the Mann–Whitney U test for independent groups). The effect of therapy applied on cardiac performance, angiotensin profile, and on the ratio of Ang-(1–7) to Ang II and to Ang (II + III) among Tgαq*44 groups was estimated by one-way ANOVA followed by Tukey’s post hoc test. If the normality assumptions were not fulfilled, the nonparametric Kruskal–Wallis test was used followed by Dunn’s post hoc test.

## Results

### Effect of Spontaneous Running, Angiotensin-Converting Enzyme Inhibition Treatment, or Their Combined Effects on the ACE/ACE-2 Balance in the Murine Model of Heart Failure at the Advanced Stage

**FIGURE 1:**
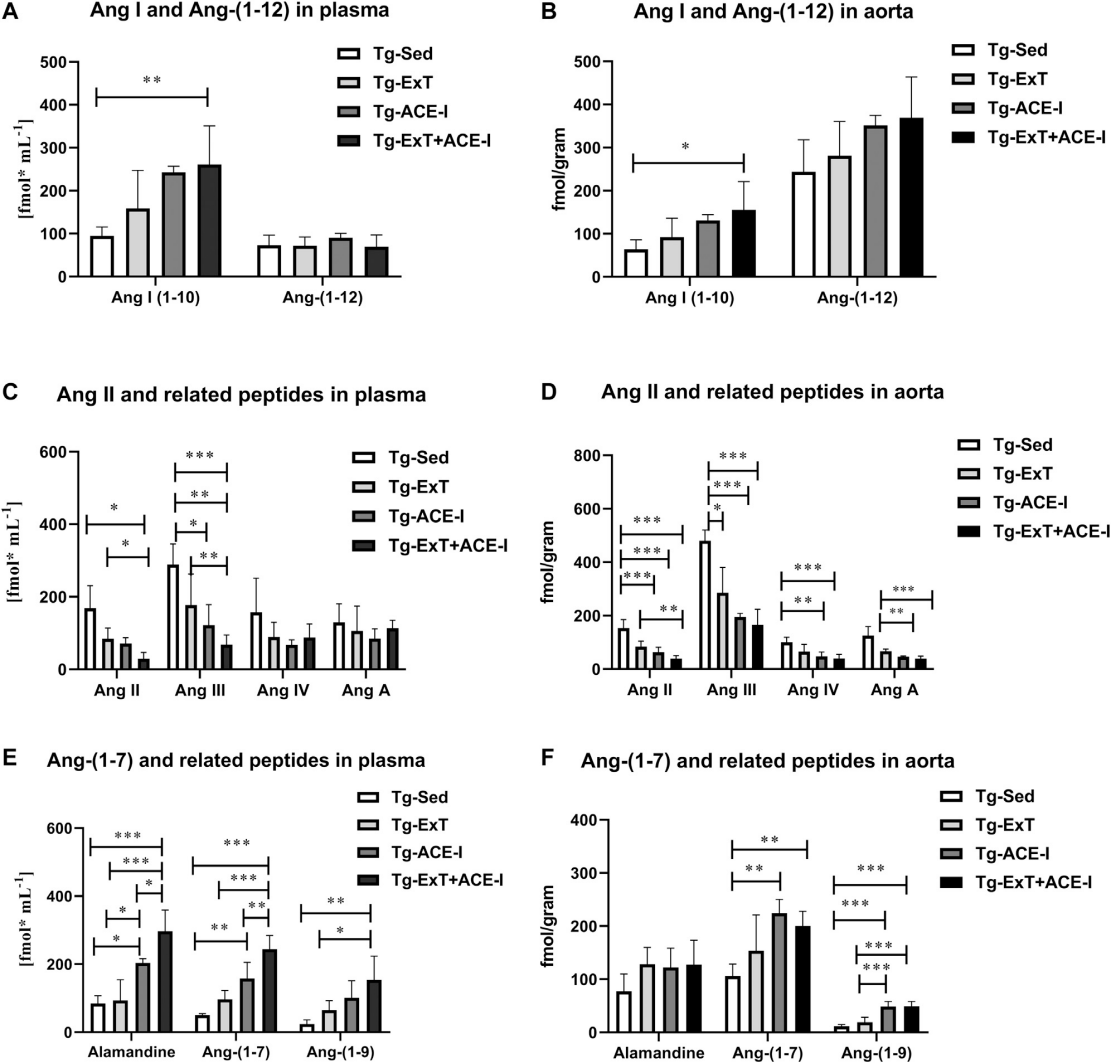
Plasma and aortic angiotensin profile in exercise-trained, ACE-I–treated, or after applied combined therapy in Tgαq*44 mice as compared to nontreated and sedentary Tgαq*44 mice. All applied therapies improved the ACE/ACE-2 balance of the RAAS, decreasing concentration of Ang II and III **(C and D),** and increasing the concentration of alamandine, Ang-(1–7), Ang-(1–9) **(E and F)**, and Ang-(1–10) **(A and D)**. An increased level of Ang-(1–12) in the aorta **(B)** but not in plasma **(A)** was also observed. Values were presented as mean ± SEM; *n* = 4–9. *p < 0.05, **p < 0.01, and ***p < 0.001. Statistics: one-way ANOVA or nonparametric Kruskal–Wallis test between Tg-ExT, Tg-ACE-I, Tg-Sed, and Tg-Exc+ ACE-I for each angiotensin.

Spontaneous running or ACE-I—either of the approaches—resulted in favorable changes in angiotensins’ profile in plasma in Tgαq*44 mice (Figures 1A,C,E). In particular, both spontaneous running and ACE-I decreased plasma Ang II and III concentration and combined therapy induced further decrease in plasma Ang II and III concentration. An increase in plasma Ang-(1–7) concentration induced by ACE-I therapy was more pronounced as compared with ExT, while a decrease in plasma Ang II concentration was similar after ACE-I and ExT. Despite these differences, the most powerful effect was observed after combined therapy as compared to sedentary and exercised/nontreated Tgαq*44 mice. Ang-(1–7)/Ang II ratio in plasma increased in the combined treatment group (ExT + ACE-I) by 11.19 ± 6.84 as compared to ACE-I (2.40 ± 1.21) or ExT alone (1.19 ± 0.18), clearly suggesting additive favorable effects on ACE-2/Ang-(1–7) and ACE/Ang II axes’ balance induced by a combination of ACE-I with ExT (Table 1). Similar results were achieved when ratio of Ang-(1–7)/Ang II + Ang III was calculated (Table 1).

**TABLE 1:** Plasma and aortic ratio of Ang-(1–7) to Ang II reflecting the activity of classical and counter-regulatory axes of RAAS.

	Tg-Sed	Tg-ExT	Tg-ACE-I	Tg_ExT + ACE-I
Plasma				
Ang-(1–7)/Ang II	0.34 ± 0.15	1.187 ± 0.18*	2.40 ± 1.20	11.19 ± 6.83*^†#^
Ang-(1–7)/(Ang II + AngIII)	0.11 ± 0.019	0.04 ± 0.14	0.98 ± 0.60	2.99 ± 1.62*^†^
Aorta				
Ang-(1–7)/Ang II	0.72 ± 0.27	1.82 ± 0.61	3.71 ± 0.73	5.55 ± 1.18*^†^
Ang-(1–7)/(Ang II + AngIII)	0.16 ± 0.03	0.4 ± 0.16	0.87 ± 0.042*^†^	1.05 ± 0.32*^†^

The mean values of Ang-(1–7)/Ang II significantly increased in plasma and aorta of Tgαq*44 mice subjected to combined therapy as compared to sedentary or exercised Tgαq*44 mice as well as increased in plasma of Tgαq*44 mice with applied combined therapy as compared to ACE-I–treated mice. Values were presented as mean ± SD; *n* = 4–9. *p < 0.05 as compared to Tg_Sed and ^†^ *p* < 0.05 as compared to Tg_ExT, ^#^ *p* < 0.05 as compared to Tg_ACE-I. Statistics: one-way ANOVA or nonparametric Kruskal–Wallis test between different Tgαq*44 experimental groups (Tg-ExT, Tg-ACE-I, Tg-Sed, and Tg-Exc+ACE-I).

Plasma concentration of alamandine and Ang-(1–9) changed parallel to plasma Ang-(1–7) (Figure 1E), while Ang III mirrored changes of Ang II (Figure 1C). Other angiotensins such as Ang-(1–12), Ang IV, and Ang A did not change significantly (Figures 1A,C), while Ang I increased rapidly in response to either ACE-I or ExT without an additive effect in combined therapy involving both of them (Figure 1A).

Both the spontaneous physical activity and the ACE-I increased Ang-(1–7) content in the aorta and decreased Ang II level (Figures 1D,F). Although the changes in Ang-(1–7) were not clearly higher after combined ACE-I and ExT as compared with ExT alone, the decrease in Ang II content in the aorta in response to combined treatment (ACE-I and ExT) was more intense than ACE-I or ExT alone (Figures 1D,F). Furthermore, the ratio of Ang-(1–7) to Ang II in Tgαq*44 mice subjected to a combined therapy with ACE-I and ExT (5.56 ± 1.79) increased to a higher degree than exercise (1.82 ± 0.61) or pharmacotherapy given alone (3.72 ± 0.73) (Table 1), suggesting additive favorable effects of combined treatment on the Ang-(1–7)/Ang II balance in the aorta, similar to the effect observed in plasma.

### Effect of Spontaneous Running, Angiotensin-Converting Enzyme Inhibition Treatment, or Their Combined Effects on Cardiac Performance in the Murine Model of Heart Failure at the Advanced Stage and Its Development

**FIGURE 2:**
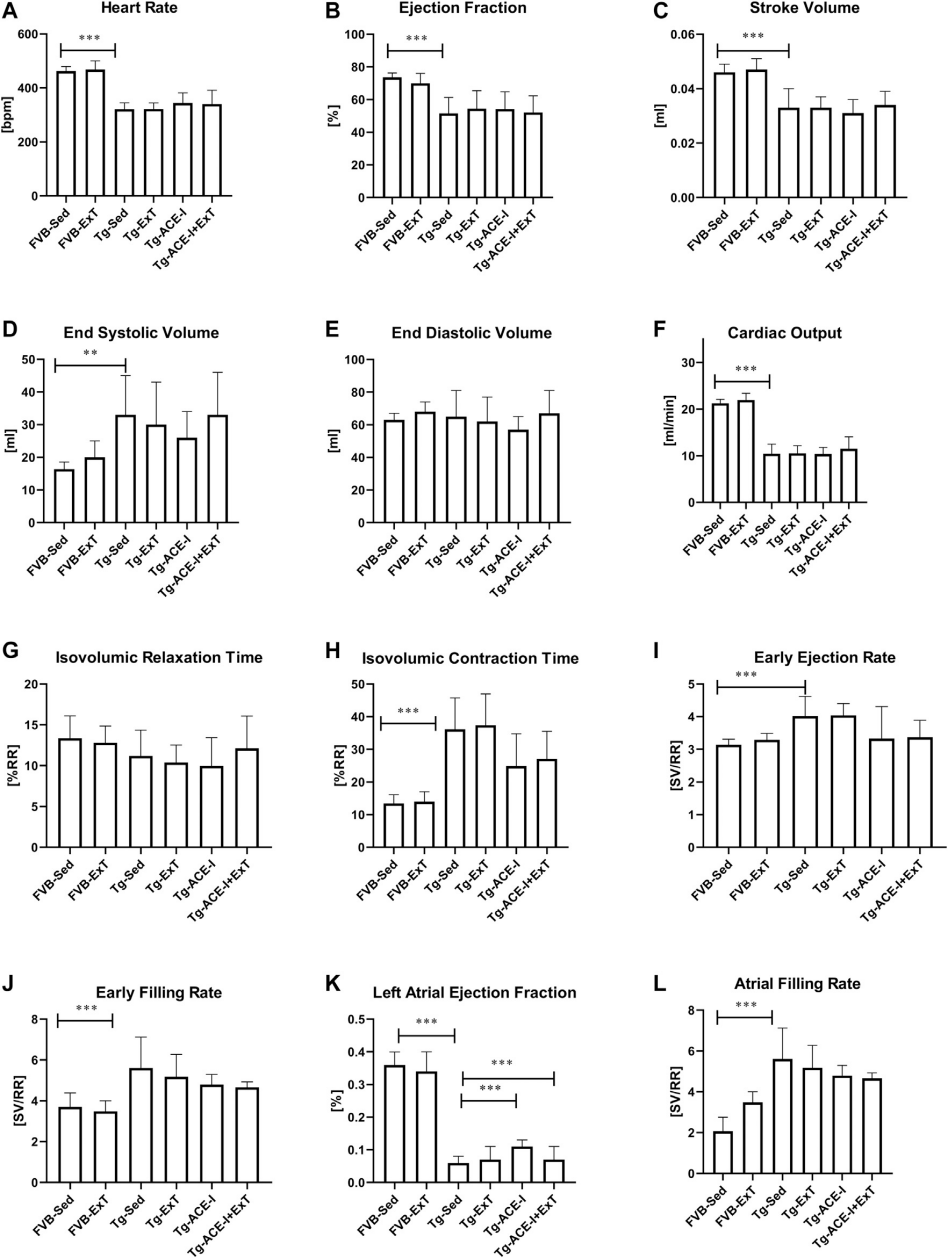
Cardiac function in FVB and Tgαq*44 mice at the end-stage of HF after 8 weeks of treatment [ExT or ExT+ therapy (ACE-I)]. The differences between FVB controls and Tgαq*44 mice (nonrunning and nontreated) were observed in heart rate **(A)**, ejection fraction **(B)**, stroke volume **(C)**, end systolic volume **(D)**, cardiac output **(F)**, isovolumic contraction time **(H)**, early ejection and filling rate **(I, J)**, and atrial filling rate **(L)**. There was a difference in cardiac performance between Tgαq*44 mice with applied ACE-I therapy and sedentary Tgαq*44 mice in left atrial ejection fraction **(K)**. Further, there was a difference in left atrial ejection fraction between sedentary Tgαq*44 mice with applied ACE-I therapy and exercised, nontreated Tgαq*44 mice **(K)**. No other alterations were observed between sedentary and exercised mice (treated or not) from each group **(E, G)**. Sed-sedentary; ExT-exercised; data presented as mean ± SD; *n* = 7–12. **p < 0.01, ***p < 0.001 for Tgαq*44 (Sed, control) *vs.* FVB (Sed, control) (*t* test or Mann–Whitney test). ^†^ *p* < 0.05 for Tgαq*44 (Sed, control) *vs.* Tgαq*44 (Sed, therapy), ^‡^ *p* < 0.05 for Tgαq*44 (Sed, control) *vs.* Tgαq*44 (ExT, therapy), ^$^ *p* < 0.05 for Tgαq*44 (Sed, therapy) *vs.* Tgαq*44 (ExT, control). Statistics within Tgαq*44 experimental groups: one-way ANOVA or Kruskal–Wallis test.

**FIGURE 3:**
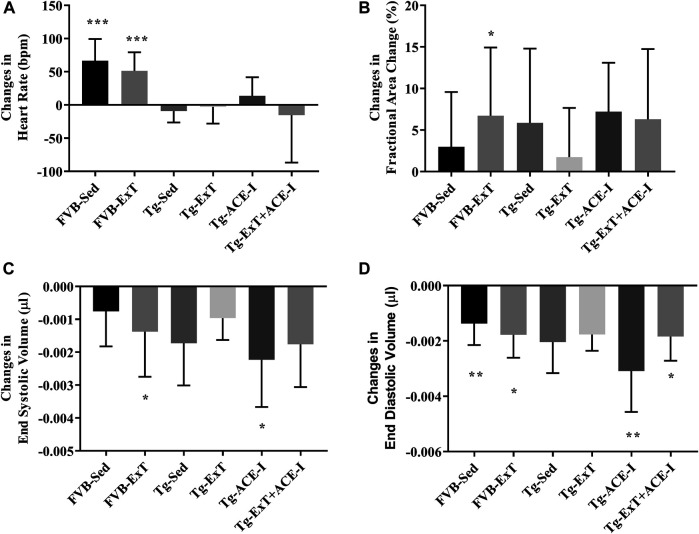
Cardiac reserve under dobutamine stimulation. Chronotropic response to dobutamine was observed in healthy controls, while no chronotropic acceleration was noted in Tgαq*44 mice at the measured stage of HF **(A)**. Cardiac inotropic response to dobutamine was observed in FVB-exercised (as increased FAC) and increased contractility to stimulation was noticed in FVB-exercised and sedentary Tgαq*44 mice with applied therapy **(B, C)**. EDA profoundly decreased after stimulation in sedentary and exercised FVB mice and in sedentary and exercised Tgαq*44 mice with applied therapy **(D)**. Values were presented as mean ± SD; *n* = 6–12. *p < 0.05, **p < 0.01, and ***p < 0.001 for dobutamine stimulation *vs.* basal condition. Statistics: *t* test for comparisons between basal values as compared to values obtained under dobutamine stimulation for each parameter.

Neither ExT, ACE-I treatment nor combined therapies influenced cardiac function in Tgαq*44 mice at the advanced stage of HF development (Figure 2). Heart rate (HR), end systolic volume (ESV), end diastolic volume (EDV), stroke volume (SV), ejection fraction (EF), cardiac output (CO), cardiac index (CI) did not change as compared to sedentary and nontreated Tgαq*44 groups. Dobutamine-induced stress test uncovered lack of chronotropic reserve in all experimental groups of Tgαq*44 mice (some of them even slowed down their rhythm in response to dobutamine) as compared to well-preserved chronotropic reserve in FVB controls (Figure 3A). The cardiac contractility reserve observed as fractional area change (FAC) increase was noticed in sedentary Tgαq*44 mice as well as in Tgαq*44 mice after ACE-I treatment (with and without ExT) and in FVB subjected to ExT but did not reach statistical significance in all Tgαq*44 groups (Figure 3B). ESV decreased in response to dobutamine in ACE-I–treated Tgαq*44 mice and in exercised FVB mice, whereas EDV decreased in all FVB groups and in Tgαq*44 mice after ACE-I treatment (with and without ExT) (Figures 3C,D).

## Discussion

The present study demonstrated that ACE-I treatment combined with spontaneous physical activity had an additive beneficial effect on the balance between vasoprotective/cardioprotective ACE-2/Ang-(1–7) and detrimental ACE/Ang II pathways in plasma and in the aorta in the advanced stage of HF in Tgαq*44 mice. These results suggest that Ang-(1–7)/Ang II balance represents a good biochemical end point to assess therapeutic value of combination of exercise with ACE blockade. However, additive changes in ACE/ACE-2 balance in plasma and tissue induced by spontaneous physical activity and ACE-I treatment in Tgαq*44 mice did not result in the improvement of failing heart function most likely due to the advanced stage of the disease.

Profound improvement in RAAS balance by combined therapy in regard to the main RAAS angiotensins [Ang II and Ang-(1–7)] as well as to other known peptide, playing role in systemic/tissue/cellular physiology [alamandine, Ang-(1–9), Ang III, and Ang IV] probably resulted from the increased ACE-2 expression and decreased ACE expression in response to exercise (Pereira et al., 2009; Kar et al., 2010; Fernandes et al., 2011; Gomes-Santos et al., 2014; Nunes-Silva et al., 2017) and from the ACE inhibition by perindopril (Ocaranza et al., 2006; Huang et al., 2010). Above exercised-induced changes are potentially regulated by changes in microRNAs (Pereira et al., 2009; Fernandes et al., 2011; Barretti et al., 2012; Martinelli et al., 2014). In consequence, there was a fall in Ang II production and activation of synthesis of Ang-(1–7). The lower Ang II level and its metabolites (Ang III and Ang IV) and subsequently diminished AT_1_R activation and simultaneously increased Ang-(1–7) and alamandine and subsequent activation of their receptors (MasR and MrgD receptors, respectively) should have led to blunting of the detrimental effects and the stimulation of vasoprotective and cardioprotective effects of RAAS. Unmetabolized Ang I may have been converted to Ang-(1–9), another cardioprotective peptide. Moreover, exercise can reduce ACE-2 shedding (Somineni et al., 2014) and can lead to an increase in the ACE-2 activity in the tissue. Physical training may also increase protein expression for Mas receptors and mRNA (Filho et al., 2008; Silva et al., 2011; Shah et al., 2012; Gomes-Santos et al., 2014), which in turn may stimulate increase of Ang-(1–7).

The above scenario of angiotensins profile shift based on the existing literature (Nunes-Silva et al., 2017) could result in the improvement in quality of life, significant vasoprotective effects, and a decreased mortality in HF. In this context, systemic effects mediated by combined therapy can greatly outweigh the effects of therapy on cardiac performance that latter not always observed in previous reports (Sullivan et al., 1988). In our study, somewhat similar to many previous experimental studies in advanced HF, we did not observe the improvement in cardiac function after ACE-I or ExT when applied alone (Sullivan et al., 1988; Belardinelli et al., 1999; van Tol et al., 2006; Felix et al., 2018), but we did not study the survival or peripheral vascular function. Of note, peripheral endothelial dysfunction has prognostic and therapeutic significance in HF. On the other hand, cardioprotective effects of ExT alone or treatment alone on cardiac performance were observed in our previous studies in early and transition stages of HF in Tgαq*44 mice (Wozniak et al., 2013; Bardi et al., 2019). Although the improvement in quality of life in HF patients may have occurred without changes in cardiac performance (van Tol et al., 2006), we cannot refer to this aspect of HF pathophysiology in a preclinical study carried out in mice. Yet, we cannot exclude that applied therapies may have improved vascular function, even though profoundly deteriorated cardiac function and structure did not respond to applied therapies.

Nevertheless, the important finding of this study was to show improved ACE/ACE-2 balance that may have a critical influence on many important factors modulating HF progression (Santos et al., 2006) and thus may represent a useful biomarker indicative of effectiveness of the therapy used.

In our recent study (Tyrankiewicz et al., 2018), we demonstrated that HF progression in this murine model of HF was associated with pronounced activation of the local ACE/Ang II pathway that was counterbalanced by prominent ACE-2/Ang-(1–7) activation. The early alterations in ACE/ACE-2 pathways were observed between 6 and 8 months and were associated with the deterioration in cardiac performance (decrease in ejection fraction) and acceleration of cardiac fibrosis. Based on these results, we suggested that the dominance of ACE-2/Ang-(1–7) over ACE/Ang II in the adaptive stage of HF may contribute to the late onset of apparent cardiac dysfunction in this model and the balance between ACE/Ang II and ACE-2/Ang-(1–7) in favor of the first axis determines the progression to the end-stage of HF.

Our results in the present work extend our previous findings and suggest for the first time that ACE-I treatment combined with spontaneous physical activity had an additive beneficial impact not only on the suppression of the RAAS–ACE/Ang II pathway in plasma and in the aorta but also on the activation of the counter-regulatory arm of the ACE-2/Ang-(1–7) pathway. These results are in line with unequivocal evidence that Ang-(1–7) has vasoprotective, cardioprotective, and anti-inflammatory effects that could contribute to inhibition of HF progression (Kassiri et al., 2009; Patel et al., 2016) (Mas receptor) (Santos et al., 2006).

That additive effect on Ang-(1–7)/Ang II ratio was noted in different physiological/cellular compartments (plasma and aorta), suggesting that even though systemic and local pathways might be regulated independently (Dell’Italia et al., 1997), the net effect of combined ACE-I and ExT was similar. Of note, the effect of combined ACE-I and ExT was quite robust and was noted not only as the downregulation/upregulation of the main components of the RAAS [Ang II, (1–7)] but was also visible as evident changes in their downstream metabolites Ang III, Ang A, and alamandine—the latter with proven vasoprotective activity exerted *via* MrgD receptor stimulation (Villela et al., 2014).

It is important to add that the amount of the spontaneous physical activity freely undertaken by the Tgαq*44 mice might have been not sufficient to meet the “threshold value” required to exert the expected improvement of the heart function in the studied mice. This could be caused by limited exercise capacity of the Tgαq*44 mice and reduced ability to undertake the spontaneous physical exercise due to the advanced heart known to affect functioning of several body organs including the brain (Adamski et al., 2018; Doehner et al., 2018; Wang et al., 2019). Furthermore, exaggerated sympatho-excitation (Fischer et al., 2005) present in the Tgαq*44 mice, as reported previously (Tyrankiewicz et al., 2013), can itself limit the exercise tolerance and contribute to the progression of the HF in the Tgαq*44 mice (Wang et al., 2019). In the light of these conclusions, it seems justified to try in the future pharmacological treatment with varied doses of forced controlled exercise interventions to establish the minimal amount of physical work load required to induce an improvement in the heart function of the pharmacologically treated patients.

## Conclusion

Taken together, our findings show that ACE-I treatment combined with spontaneous physical activity had an additive effect in increasing activity of the ACE-2/Ang-(1–7) pathway and in decreasing activity of ACE/Ang II. These results might be of high pathophysiological/therapeutic importance as ACE/ACE-2 balance is one of the key factors regulating HF progression, patients’ survival, and quality of life. Accordingly, Ang-(1–7)/Ang II ratio should be further exploited as a mechanistically-relevant biomarker in guided therapy of HF and could contribute to optimized exercise combined with pharmacotherapy to afford improved outcomes in patients with HF.

## Data Availability

The raw data supporting the conclusions of this article will be made available by the authors without undue reservation.
